# NEW APPROACH METHODOLOGIES (NAMS) TO STUDY AGING

**DOI:** 10.1093/geroni/igae098.0037

**Published:** 2024-12-31

**Authors:** Tiziana Cogliati, Jennifer Fox

**Affiliations:** NIA-NIH, Bethesda, Maryland, United States; NIA-NIH, Bethesda, Maryland, United States

## Abstract

New Approach Methodologies (NAMs) include human-relevant cell-based (in vitro), cell-free (in chemico), and computational-based (in silico) models that are complementary, not a replacement, to existing animal models. This workshop focuses on 3-dimentional (3-D) in vitro human models including tissue chips, organoids, explants, known as microphysiological systems (MPS). The adoption of human in vitro NAMs for basic and translational research is expanding, recently promoted by the FDA Modernization Act 2.0 that authorizes their use to investigate drug safety and efficacy. Extending the use of human in vitro NAMs to the study of aging and the development of geroprotective interventions presents challenges recognized by the research community. These challenges can be addressed by leveraging our knowledge of the biology of aging and fostering interdisciplinary collaborations. This interdisciplinary session is designed to educate basic scientists, physician-scientists, clinicians, and a general audience of industry representatives interested in adopting NAMs for aging research. One of the goals is to promote a dialog about the challenges and opportunities posed by human in vitro NAMS and inspire new collaborative research initiatives bringing together basic scientists, bioengineers, physician-scientists and clinicians, and members of other GSA sections. At the conclusion of the session, attendees will be able to: (1) describe the different types of human in vitro NAMs and their applications in basic and translational research; (2) explain the challenges and current solutions to adopting human 3-D in vitro NAMs in research on aging; and (3) discuss how data from 3-D in vitro NAMs and from animal models are complementary.

